# Essential oils of *Psidium cattleianum* Sabine leaves and flowers: Anti-inflammatory and cytotoxic activities

**DOI:** 10.3389/fchem.2023.1120432

**Published:** 2023-02-06

**Authors:** Heba E. Elsayed, Eman M. El-Deeb, Heba Taha, Hussein S. Taha, Mohamed R. Elgindi, Fatma A. Moharram

**Affiliations:** ^1^ Pharmacognosy Department, Faculty of Pharmacy, Helwan University, Cairo, Egypt; ^2^ Pharmacognosy Department, Faculty of Pharmacy, October 6 University, Giza, Egypt; ^3^ Biochemistry and Molecular Biology Department, Faculty of Pharmacy, Helwan University, Cairo, Egypt; ^4^ Department of Plant Biotechnology, Genetic Engineering Division, National Research Centre, Cairo, Egypt

**Keywords:** anti-inflammatory, caryophyllene, cytotoxicity, MCF-7, *Psidium cattleianum*, supercritical fluid extraction

## Abstract

**Introduction:** *Psidium cattleianum* Sabine is a Brazilian native shrub cultivated for its edible fruit araçá (strawberry guava). *P. cattleianum* is recognized for health and food applications, although the essential oils (EOs) from the Egyptian inhabitant are not fully explored. The current study investigated the anti-inflammatory and cytotoxic activities of EOs from *P. cattleianum* leaves and flowers.

**Materials and methods:** The EOs were obtained by three different methods *viz*; the conventional hydro-distillation, microwave assisted hydro-distillation, and supercritical fluid extraction, while their analysis was accomplished using GC/MS. The derived EOs were screened for their anti-inflammatory activity in the 5-lipoxygenase, COX-1, and COX-2 enzyme based assays, while the anticancer potential was deduced from MTT cytotoxic assay, cell cycle, and western blotting analysis.

**Results and discussion:** Among other methods, supercritical fluid extraction offered the highest EO yield, 0.62% (leaves) and 1.4% (flowers). GC/MS identified β-caryophyllene and α-humulene in both organs with high but variable percentages. The leaves demonstrated strong activity in inhibiting the 5-lipoxygenase enzyme (IC50 2.38), while the flowers, in inhibiting COX-2 (IC50 2.575). Moreover, the leaves showed potent, selective cytotoxicity to MCF-7 cells (IC50 5.32) via apoptosis by modulating the p53/Bax/Bcl2 axis. The deduced activities are possible due to the synergism between the volatile components that endorses *P. cattleianum* leaves’ EOs in the management of breast cancer and inflammatory disorders.

## 1 Introduction

Essential oils (EOs) are presently attracting interest in the scientific community, owing to their imperative pharmacological activities (Amorim et al., 2009). They have been traditionally used by ancient cultures as a complementary holistic health approach, which was later termed aromatherapy, as they possess an array of unique health-fostering benefits. Currently, hundreds of EOs have been identified as commercially important crude drugs in the therapeutic, horticultural, cosmetic, and food industries (Shakeri et al., 2016). In addition, their unique chemical framework is accredited to a variety of effective biological activities, such as antioxidant, antimicrobial, antinociceptive, anti-inflammatory, and anticancer activities (Amorim et al., 2009; Singh et al., 2013). To date, species of the family Myrtaceae are among the recognized plants that provide many valuable products including EOs (Arain et al., 2019). Notably, *Psidium* is one of the economically important genera because of its distinguished edible, essential oil-rich species such as *P. guajava* L. and *P. cattleianum* Sabine (Beltrame et al., 2021). *P. cattleianum* Sabine is a Brazilian native shrub, where it is commonly known as “araçá” (Faleiro et al., 2016); however, it is cultivated through the tropics and subtropics for its juicy, purple-red fruits known as strawberry guava or Cattley guava (Patel, 2012). The fruit has a strawberry-like flavor with a spicy touch and is rich in vitamin C, which is 3–4 times more than citrus fruits (Chalannavar et al., 2013). The characteristic flavor is due to the presence of essential oils (EOs), which were previously extracted by the hydro-distillation method from *P*. *cattleianum* grown in different countries and extensively studied by several researchers (Chalannavar et al., 2013; Soliman et al., 2016; dos Santos et al., 2018; Chrystal et al., 2020; Silvestre et al., 2022). On the other hand, only one report was conducted on the EOs from Egyptian species (Soliman et al., 2016). The relevant literature stated that the EOs derived from *P. cattleianum* leaves possessed significant *in vitro* antioxidant, antimicrobial, anti-inflammatory, and anticancer activities (Castro et al., 2015; Chrystal et al., 2020; de Souza et al., 2021). To the best of our knowledge, no prior study has investigated the volatile metabolites from the flowers; hence, it is interesting to investigate the chemical nature of its derived EOs in comparison with those obtained from the leaves, in an attempt, to discover a new active essential oil-based remedy for the management of cancer and inflammatory disorders.

Cancer is considered one of the most severe diseases worldwide and is expected to increase due to the lifestyle adopted nowadays (Bray and Moller., 2006). The World Health Organization (WHO) revealed that cancer was responsible for nearly 30% of deaths from non-chronic diseases among adults in 2020. Drug resistance to cancer and the toxicity of available chemotherapeutic agents are currently the main limitations in its treatment. So, the discovery of innovative and safe treatments is considered a big challenge (Abdullaev, 2001). However, this mission is not impossible with natural products. Natural products have played a pivotal role in cancer chemotherapy and chemoprevention for over half a century and established anticancer drugs, e.g., camptothecin, doxorubicin, paclitaxel, vinblastine, and vincristine (Sarker et al., 2020). Hence, the discovery of new anticancer hits derived from natural sources is a feasible strategy and is in dire need.

The functional correlation between inflammation and cancer is not new. It is now becoming clear that the cancer micro-environment, which is largely composed of inflammatory cells, is an indispensable participant in neoplastic development, encouraging proliferation, survival, and migration. COX-1 and COX-2 are two important isoforms of the cyclooxygenase family in which COX-2, the inducible isoform of COX, has developed as the key enzyme in the regulation of inflammation and cancer (Agrawal and Mishra, 2010). Other reports mentioned the disposition of COX-2 and lipoxygenases (LOX) in the regulation of different normal physiological processes and inflammation, in addition to cancer (Wang and Du Bois, 2010). These insights are raising new anti-inflammatory therapeutic approaches for the development of cancer. Natural products play a significant role in human health in relation to the prevention and treatment of inflammatory conditions witnessed by curcumin, cucurbitacins, and 1,8-cineole, in which the latter is an essential oil-derived terpene oxide (Juergens et al., 2020). Hence, searching for new anti-inflammatory active agents derived from natural products or even essential oils is a propitious approach.

In continuation to our research into the discovery of anti-inflammatory and anticancer bioactive hits from natural sources, we aimed to identify the chemical composition of the EOs obtained from *P. cattleianum* leaves and flowers cultivated in Egypt for the first time using three different techniques. Moreover, it was deemed of interest to validate their cytotoxicity potential in different cancer cell lines and unravel their detailed underlying mechanisms in terms of cell cycle analysis and apoptosis-related proteins. Furthermore, the inhibitory activities of the derived EOs to 5-LOX, COX-1, and 2 were investigated to expand the conception that inflammation may be a serious factor in cancer progression.

## 2 Material and methods

### 2.1 Plant material

Both leaves and flowers of *Psidium cattleianum* Sabine were collected at the fruiting stage from March to April 2021 from Mazhar Botanic Garden, Cairo, Egypt. The plant was identified by Dr. Trease Labib, Senior Botanist at Mazhar Botanic Garden, Cairo, Egypt. Plant voucher samples (01Pca/2021) were represented at the herbarium of Pharmacognosy Department, Faculty of Pharmacy, Helwan University, Cairo, Egypt.

### 2.2 Extraction of essential oils of flowers and leaves

#### 2.2.1 Conventional hydro-distillation method

Small pieces of fresh leaves (200 g) and flowers (50 g) were mixed with double distilled water before hydro-distillation (HD) using Clevenger apparatus for 4 h.

#### 2.2.2 Microwave-assisted hydro-distillation method

The microwave-assisted hydro-distillation (MAHD) was achieved using a microwave oven (CEM Corporation, Matthews, NC, United States) and a model (MARS 240/50, No. 907511, 1,200 W) operated at a frequency of 2,450 MHz. In brief, small pieces of fresh leaves (200 g) and flowers (50 g) were placed in 1L- and 500-mL flask and mixed with 500 and 250 mL deionized water, respectively. After that, the Clevenger apparatus was set up within the microwave oven cavity, while the cooling system connected to the outside of the oven to condense the distillate volatiles continuously. The microwave oven was operated at an 800-W power level for 60 min (Ghazanfar et al., 2020).

#### 2.2.3 Supercritical fluid extraction

The supercritical fluid extraction (SFE) using supercritical carbon dioxide was accomplished as per the procedure described by Suetsugu et al. (2013) using Speed TM SFE-2/4, applied separations, and constructed in conjunction with the USDA1-USA. About 200 g and 50 g of dried and milled leaves and flowers were extracted at 40°C and 15.0 MPa. First, the apparatus was operated in a static mode for 60 min and then in dynamic mode for 60 min with a final total processing time of 180 min. The main drawback of SCE is its low polarity; this problem is overcome by employing polar modifiers by the addition of absolute ethanol with a flow rate of 0.2 mL/min as a co-solvent to alter the polarity and increase its solvating power. The oil obtained from the three methods was dried in anhydrous Na_2_SO_4_ and stored in amber, sealed bottles at 4°C until GC/MS analysis.

Moreover, the oil percentage was calculated as an essential oil volume (mL)/100 g of fresh plant material.

### 2.3 Gas chromatography coupled with mass spectrometry analysis

Gas chromatography-mass spectrometry (GC/MS) investigation was conducted using Shimadzu GCMS-QP2010 (Kyoto, Japan) coupled with quadrupole mass spectrometer (Shimadzu Corporation, Kyoto, Japan). Separation of oil compounds was achieved using an Rtx-5MS column (30 m × 0.25 mm i.d. × 0.25-µm film thickness, Restek, United States) with a flame ionization injector. The temperature of the column was kept at 50°C for 3 min in the beginning (isothermal), then planned to increase it to 300°C at a rate of 5°C/min, and then continuously kept for 10 min at 300°C (isothermal). The temperature of the injector was adjusted to 280°C. The flow rate of the helium carrier gas is 1.37 mL/min for HD and MAHD samples, while that of SCE sample is 1.41 mL/min. The mass spectra were recorded as follows: the temperatures of the interface and ion source are 280°C and 200°C, respectively; the mode of electron ionization is 70 eV with a scanning range of 35–500 amu. The split mode (1: 15) was used for injecting the oil samples (1 μL).

### 2.4 Identification of volatile oil components

The obtained volatile constituents were identified by comparing their Kovats retention indices (RI) with that of standard *n*-alkane series (C_8_–C_28_) and their mass spectra with those reported in the NIST (National Institute of Standards and Technology) and Wiley mass spectral databases (similarity index >90%) (Adams, 2005).

### 2.5 *In vitro* biological evaluation

All *in vitro* assays were accomplished on the EOs isolated from *P. cattleianum* leaves and flowers using the SFE method as this extraction method offered the highest oil yield.

#### 2.5.1 Enzyme-based anti-inflammatory assays

##### 2.5.1.1 5-Lipoxygenase inhibitory screening assay

It was performed using a BioVision 5-lipoxygenase inhibitor screening assay kit (catalog no. K980-100; Milpitas, CA, United States). Four concentrations of the EOs (0.1, 1, 10, and 100 μL/m) were prepared in DMSO (Sigma-Aldrich, Steinheim, Germany). Then, 2 μL of each EO stock solution, 5-LOX enzyme (enzyme control), DMSO (negative control), or zileuton (positive control) were added separately to a 96-well plate; then, 38 μL of LOX buffer was added. Subsequently, 40 μL of the reaction mixture (34 μL LOX buffer, 2 μL LOX probe, and 4 μL 5-LOX enzyme) was added to each well except for the enzyme control well, which will receive a reaction mixture composed of 38 μL LOX buffer and 2 μL LOX probe only. The plate was incubated for 10 min at room temperature before the addition of 20 μL of 5% LOX substrate (prepared in LOX buffer) to each well. The experiment was performed recurrently in triplicate, and the percentage inhibitions of EOs were measured fluorometrically using the following equation:

% Inhibition = [(slope of enzyme control−slope of tested EO)/slope of enzyme control] x 100.

IC_50_ represents the EO concentration that causes 50% enzyme inhibition from the dose-response curve using non-linear regression analysis.

##### 2.5.1.2 COX-1 and COX-2 inhibitory screening assays

It was accomplished using BioVision COX-1 and 2 inhibitor screening assay kits (catalog no K548-100 and K547-100, respectively; Milpitas, CA, United States), according to the manufacturer’s instructions. Briefly, four different concentrations of EOs (0.1, 1, 10, and 100 μL/mL) were prepared in DMSO. Thereafter, in a 96-well plate, 10 μL of each EO stock solution or assay buffer was added separately to the wells that were assigned as sample screen [SC] and enzyme control [EC], respectively. Thereafter, 80 μL of the reaction mixture (76 μL COX assay buffer, 1 μL COX probe, 2 μL COX cofactor, and 1 μL COX-1) was added to each well, and then, 10 μL of arachidonic acid/NaOH was added. SC560 (COX-1 inhibitor), celecoxib (COX-2 inhibitor), and indomethacin (non-steroidal anti-inflammatory drug) were considered as standard control drugs. The inhibition percentage of tested EOs was measured fluorometrically and calculated as follows:

% Inhibition = [(slope of EC–slope of SC)/slope of EC] x 100.

IC_50_ represents the concentration of EOs that causes 50% enzyme inhibition deduced from the dose-response curve using non-linear regression analysis.

#### 2.5.2 Anticancer assays

##### 2.5.2.1 Cell lines and culture conditions

The human hepatocellular (HepG2), breast (MCF-7), and immortalized myelogenous leukemia (K562) cancer cell lines and the normal fibroblast lung cells (WI-38) were supplied from the company for biological products and vaccines (VACSERA, Egypt). The HepG2 and MCF-7 cells were sustained in Dulbecco’s modified Eagle’s medium (DMEM), K562 in Roswell Park Memorial Institute medium (RPMI-1640), and WI-38 were preserved in Eagle’s Minimum Essential Medium (EMEM). All media were supplemented with 10% fetal calf serum (FCS), 2 mM glutamine, 100 U/mL penicillin, and 100 μg/mL streptomycin at 37°C at 5% CO_2_ (v/v) atmosphere.

##### 2.5.2.2 Cytotoxicity assay

Cell viability was evaluated using the MTT reagent (3-(4, 5-dimethyl thiazolyl-2)-2,5-diphenyltetrazolium bromide, Sigma–Aldrich, Steinheim, Germany) colorimetric assay (Papadimitriou et al., 2019). Briefly, MCF-7, HepG2 and K562, and WI 38 cell lines were added to a 96-well culture plate (1.2–1.8 x 10^3^ cells/well). The cells were incubated for 24 h and then treated with an increasing concentration of tested EOs (3.125, 6.25, 12.5, 25, and 100 μL/mL) for 48 h. Then, the supernatant of the culture was removed followed by the addition of 40 µL of an MTT solution. The formazan crystals of MTT were dissolved by the addition of DMSO (180 µL). Finally, color absorbance was measured using a microplate reader at λ_570_ nm (Sunrise, TECAN, Inc, United States), and doxorubicin^®^ (Sigma Company, Suffolk County, NY, United States) was used as a standard cytotoxic agent. Triplicate repeats were performed, and the cell viability percentage was measured as follows:

Cell viability percentage = (treated cell absorbance/control cell absorbance) x 100.

IC_50_ was calculated by non-linear regression analysis.

##### 2.5.2.3 Cell cycle analysis

Since the EO of *P. cattleianum* leaves displayed potent cytotoxic activity toward the MCF-7 cell line, the following assays were carried out to extensively evaluate its mode of cytotoxicity and identify the downstream signaling pathway. So, to assess the leaves' EO effects on MCF-7 cell distribution *via* different stages of the cell cycle, the DNA content of the propidium iodide (PI)-stained nuclei was evaluated by flow cytometry, according to Ormerod (1994). The cells were treated with a dose equivalent to the IC_50_ of the EOs of the leaves (IC_50_ 5.32 μL/mL) for 48 h, followed by washing with ice-cold phosphate-buffered saline (PBS) twice and then collected by centrifugation. Then, the cell pellets were mixed with ethanol (75%, −20°C) and stained with the kit of PI flow cytometry (ab139418, Abcam, United States), according to the manufacturer’s instructions. Cell cycle distribution was recognized using a FACSCalibur flow cytometer (BD Biosciences, San Jose, CA) and measured using CellQuest software (Becton Dickinson Immunocytometry Systems, San Jose, CA).

##### 2.5.2.4 Apoptosis assay

Necrosis cell populations and early and late apoptosis were measured using the annexin V-FITC/PI apoptosis/necrosis kit (Cat No: K101; BioVision, Inc., United States) to evaluate the effects of leaves’ EOs on programmed cell death. Briefly, MCF-7 cells were treated for 48 h with the IC_50_ concentration of EOs of leaves (5.32 μL/mL) and then collected by trypsinization, washed twice with ice-cold PBS, re-suspended in annexin V-binding buffer (500 μL), and finally, 5 μL of annexin V-FITC was added and incubated in the dark at 25°C for 10 min. The investigation was achieved by using a FACSCalibur flow cytometer and CellQuest software.

##### 2.5.2.5 Western blot analysis

MCF-7 cells were seeded, cultured, and treated with IC_50_ of the EOs of leaves (5.32 μL/mL) for 48 h. Cell protein lysates were prepared by radio immunoprecipitation assay buffer (RIPA buffer, Cell Signaling, Danvers, MA), and the concentration of the total protein in the supernatant was measured calorimetrically using the Bradford method (Sambrook et al., 1989) before Western blot, which was assessed by mixing and boiling equal amounts of protein samples (20 µg) with sodium dodecyl sulfate (SDS) buffer for 10 min, cooled on ice, loaded into SDS polyacrylamide gel, and then separated by electrophoresis (Cleaver, United Kingdom). Then, the bands were transported by semi-dry electroblotting (Bio-Rad, United States) at 2.5 A and 25 V for 30 min to the polyvinylidene fluoride (PVDF) membranes (Bio-Rad, United States) which were blocked with non-fat dry milk in TBS-T (5%) for 2 h and incubated with anti-Bax, anti-Bcl-2, anti-p53 (Cell Signaling Technology, Inc. United States), and anti-*β*-actin (Sigma–Aldrich, United States) antibodies (1:1000) overnight, and washed with TBS-T (three times), followed by incubation with horseradish peroxidase (HRP)-linked secondary antibody (1:5000) for 1 h. Progress was carried out using a chemiluminescent ECL substrate (Perkin Elmer, United States), following the manufacturer’s recommendation, and chemiluminescent signals were taken using a CCD camera-based imager, and the intensities of bands were measured using Image Lab (Bio-Rad, United States).

#### 2.5.3 Statistical analyses

All experimental data were obtained from three separate experiments performed in three replicates. Data were expressed as the mean ± SD in both *in vitro* cytotoxic and *in vitro* anti-inflammatory assays. GraphPad Prism version 5.0 (GraphPad Software, San Diego, CA, United States) was used to calculate mean inhibitory concentrations and IC_50_ values using non-linear regression analysis.

## 3 Results and discussion

Essential oils are odorous products of complex compositions, obtained from natural sources by various methods. They possess the characteristic taste and odor of the source from which it was derived. Such organoleptic properties are principally dependent on chemical composition, which is greatly affected by intrinsic and extrinsic factors (Dhifi et al., 2016). The intrinsic factors include, but are not limited to, the plant organ, genetics, and maturity stage, while the extrinsic factors include the extraction methods and environmental conditions (Dhifi et al., 2016). In the present study, the variations in *P. cattleianum* EOs cultivated in Egypt were comparatively investigated in terms of different plant organs and extraction methods. Herein, the leaves and flowers were extracted for the first time using three different methods, namely, HD, MAHD, and SFE. The HD method is the most common and low-priced method, although the obtained oil may undergo saponification, polymerization, and/or isomerization, especially for its labile components (Koedam et al., 1979). Meanwhile, MAHD and SFE represent one of the most applied green technology and environmentally friendly methods that produce good EOs in a little time with slight environmental degradation (Abbas et al., 2022). However, the SFE method is privileged by its diffusion coefficient, high oil yield, and low oil viscosity. Hence, it is obvious that each extraction method differs in its basic principle and adjusted conditions, which subsequently affect the yield and physical and chemical composition of the obtained volatile. By the aforementioned information, the EOs derived from the leaves and flowers by HD were almost pale-yellow liquids with a fruity, acidic flavor, while those obtained from SEF exhibited brown color and fruity, aromatic flavor. Moreover, it was found that the oil’s yield was affected not only by the extraction method but also according to the plants’ organs. The measured yield was 0.14, 0.31, and 0.62 v/w (*P. cattleianum* leaves) and 0.20, 0.31, and 1.40 v/w (*P. cattleianum* flowers) for HD, MAHD, and SFE, respectively. From the obtained results, it was found that SFE corresponded to the highest oil yield, an observation that may be correlated with the unique characteristics of the SFE. SF is considered a liquid–gas intermediate phase: it is non-viscous with low or no surface tension and possesses the characteristics of both liquid and gas, which results in a great diffusion rate and solvation power, allowing faster extraction and worthy yield (Sargenti and Lancas, 1997).

### 3.1 GC/MS analysis for the extracted essential oils

The effects of the techniques used in the oil extraction from various plants’ organs were reflected in the qualitative and quantitative compositions of *P. cattleianum* EOs. Regarding, the leaves' essential oil, a total of 51 (95.57%), 50 (93.85%), and 41 (86.73%) volatile compounds were identified in HD, MAHD, and SEF oil samples, respectively (Table 1, Supplementary Figures S1–S3). Interestingly, careful interpretation of the data showed that there is no major difference among the three extraction methods in the percentage of the major identified components. For instance, it was found that humulene represented 10.9%–15.00%, *β* caryophyllene 11.77%–13.2%, germacrene B 6.02%–8.19%, and *α*-bisabolol 6.39%–7.46% in HD, MAHD, and SEF methods, respectively. On the other hand, there is a significant difference in the main EO classes as the percentage of the oxygenated compounds was 29.38, 26.65, and 31.36% for HD, MAHD, and SFE, respectively, while the percentage of non-oxygenated compounds was double the percentage of the oxygenated compounds being 66.19% (HD), 67.20% (MAHD), and 55.37% (SFE). Moreover, there is an additional exciting difference in the chemical subclass of the identified volatiles. Herein, the monoterpene (MH) and sesquiterpene (SH) hydrocarbon percentages were different among the implemented extraction methods. For instance, all oil samples displayed a low percentage of MH being 9.72, 6.05, and 0.84% in HD, MAHD, and SFE, respectively, while SH represented the highest percentage, which was calculated as 56.47% (HD), 61.15% (MAHD), and 54.53% (SFE). Concerning the oxygenated sesquiterpenes (OS), it was found that the leaves’ EOs encompass considerable contents of OS being 28.09, 25.99, and 30.8% in HD, MAHD, and SFE, respectively, in comparison with oxygenated monoterpenes (OM), which represented a very low percentage in the three extraction methods.

**TABLE 1:** Identified compounds of *P*. *cattleianum* leaves’ essential oil extracted by different methods.

Peak	R_t_	Compound	M.F.	RI_exp_	RI_lit_	%Content			Identifications
						HD	MAHD	SFE	
1	7.13	*α*-Thujene	C_10_H_16_	908	908	0.10	0.07	-	MS, RI
2	7.31	*α*-Pinene	C_10_H_16_	915	915	6.29	4.51	0.43	MS, RI
3	8.58	*β*-Pinene	C_10_H_16_	961	961	0.49	0.29	-	MS, RI
4	9.05	*β*-Myrcene	C_10_H_16_	978	978	0.19	0.06	-	MS, RI
5	10.20	D-Limonene	C_10_H_16_	1017	1017	1.22	0.61	0.23	MS, RI
6	10.51	*trans*-*β*-Ocimene	C_10_H_16_	1027	1027	0.19	0.08	-	MS, RI
7	10.83	*cis*-*β*-Ocimene	C_10_H_16_	1038	1038	1.15	0.43	0.18	MS, RI
8	11.15	*γ*-Terpinene	C_10_H_16_	1048	1048	0.09	-	-	MS, RI
10	14.84	Terpinen-4-ol	C_10_H_18_O	1167	1167	0.16	0.07	-	MS, RI
11	19.85	*α*-Cubebene	C_15_H_24_	1339	1339	-	0.07	-	MS, RI
12	20.05	*α*-Longipinene	C_15_H_24_	1346	1347	-	0.06	-	MS, RI
13	20.33	(+)-Cyclosativene	C_15_H_24_	1356	1358	-	-	0.04	MS, RI
14	20.49	Ylangene	C_15_H_24_	1362	1362	0.10	0.14	0.09	MS, RI
15	20.59	Copaene	C_15_H_24_	1365	1365	2.16	2.57	2.39	MS, RI
16	20.79	Nerol acetate	C_12_H_20_O_2_	1372	1372	1.04	0.59	0.56	MS, RI
17	21.05	*β*-Elemene	C_15_H_24_	1380	1380	0.62	0.67	0.45	MS, RI
18	21.42	7-*epi-*Sesquithujene	C_15_H_24_	1392	1391	0.15	0.20	-	MS, RI
19	21.54	*α*-Gurjunene	C_15_H_24_	1398	1398	0.20	0.28	0.17	MS, RI
20	21.64	*cis*-*α*-Bergamotene	C_15_H_24_	1402	1403	0.30	0.38	-	MS, RI
21	21.69	*trans*-*α*-Bergamotene	C_15_H_24_	1404	1405	-	-	0.31	MS, RI
22	21.90	*β* -Caryophyllene	C_15_H_24_	1414	1414	11.77	12.56	13.20	MS, RI
23	22.17	*γ*-Elemene	C_15_H_24_	1423	1423	2.38	-	2.36	MS, RI
24	22.35	Aromandendrene	C_15_H_24_	1429	1429	0.54	0.77	0.74	MS, RI
25	22.73	α-Humulene	C_15_H_24_	1445	1445	14.65	15.00	10.90	MS, RI
26	22.85	*β*-Santalene	C_15_H_24_	1449	1449	0.58	0.65	0.07	MS, RI
27	22.95	Alloaromadendrene	C_15_H_24_	1452	1452	0.44	0.58	0.55	MS, RI
28	23.08	Muurola-4,11-diene	C_15_H_24_	1458	1458	-	-	0.37	MS, RI
29	23.32	*γ*-Muurolene	C_15_H_24_	1467	1467	2.00	2.89	1.01	MS, RI
30	23.45	Germacrene D	C_15_H_24_	1473	1473	0.44	1.00	0.51	MS, RI
31	23.61	γ –Selinene	C_15_H_24_	1479	1479	1.06	1.29	3.75	MS, RI
32	23.81	γ-Maaliene	C_15_H_24_	1487	1435	1.59	1.82	-	MS, RI
33	24.09	*β*-Bisabolene	C_15_H_24_	1498	1498	1.42	2.30	2.48	MS, RI
34	24.16	*β*-Curcumene	C_15_H_24_	1501	1504	0.52	-	-	MS, RI
26	22.85	*β*-Santalene	C_15_H_24_	1449	1449	0.58	0.65	0.07	MS, RI
27	22.95	Alloaromadendrene	C_15_H_24_	1452	1452	0.44	0.58	0.55	MS, RI
28	23.08	Muurola-4,11-diene	C_15_H_24_	1458	1458	-	-	0.37	MS, RI
29	23.32	*γ*-Muurolene	C_15_H_24_	1467	1467	2.00	2.89	1.01	MS, RI
30	23.45	GermacreneD	C_15_H_24_	1473	1473	0.44	1.00	0.51	MS, RI
31	23.61	γ –Selinene	C_15_H_24_	1479	1479	1.06	1.29	3.75	MS, RI
32	23.81	γ-Maaliene	C_15_H_24_	1487	1435	1.59	1.82	-	MS, RI
33	24.09	*β*-Bisabolene	C_15_H_24_	1498	1498	1.42	2.30	2.48	MS, RI
34	24.16	*β*-Curcumene	C_15_H_24_	1501	1504	0.52	-	-	MS, RI
26	22.85	*β*-Santalene	C_15_H_24_	1449	1449	0.58	0.65	0.07	MS, RI
27	22.95	Alloaromadendrene	C_15_H_24_	1452	1452	0.44	0.58	0.55	MS, RI
46	26.46	Humulene epoxide I	C_15_H_24_O	1591	1592	0.19	0.19	0.27	MS, RI
47	26.56	Ledol	C_15_H_26_O	1595	1595	1.23	1.06	1.12	MS, RI
48	26.71	(-)-Humulene epoxide II	C_15_H_24_O	1601	1602	1.47	1.41	1.94	MS, RI
49	27.14	Di-*epi-*1,10-cubenol	C_15_H_26_O	1619	1619	1.81	1.72	2.07	MS, RI
50	27.27	Humulenol-II	C_15_H_24_O	1624	1620	1.50	1.53	-	MS, RI
51	27.37	Caryophylla-4(12),8(13)-dien-5-*α*-ol	C_15_H_24_O	1629	1632	0.67	0.69	-	MS, RI
52	27.47	*T*-Muurolol	C_15_H_26_O	1633	1634	4.09	3.39	3.73	MS, RI
53	27.52	*α*-Cadinol	C_15_H_26_O	1635	1635	1.34	1.36	3.19	MS, RI
54	28.40	*α*-Bisabolol	C_15_H_26_O	1673	1673	6.39	7.46	7.48	MS, RI
55	28.82	Eudesm-7(11)-en-4-ol	C_15_H_26_O	1692	1692	1.19	0.47	1.10	MS, RI
56	29.22	Farnesol	C_15_H_26_O	1710	1711	0.59	0.87	1.24	MS, RI
57	31.81	*trans*-Farnesyl acetate	C_17_H_28_O_2_	1824	1824	1.19	0.47	1.10	MS, RI
		Total identified compounds				95.48	93.85	86.73	
		Non-oxygenated							
		Monoterpene hydrocarbons (MH)				9.72	6.05	0.84	
		Sesquiterpene hydrocarbons (SH)				56.47	61.15	54.53	
		Oxygenated							
		Oxygenated monoterpenes (OM)				1.20	0.66	0.56	
		Oxygenated sesquiterpenes (OS)				28.09	25.99	30.8	

R_t_, retention time; RI_exp_, experimental refractive index; RI_lit_, reference refractive index; MF: molecular formula.

The EOs obtained from *P. cattleianum* flowers, which were investigated here for the first time, displayed a total of 30 (87.55%), 49 (81.96%), and 27 (68.46%) volatile compounds in the HD, MAHD, and SFE derived samples, respectively (Table 2, Supplementary Figures S4–S6). Keen analysis of the obtained data has shown that *β*-caryophyllene (18.85%), humulene (14.46), germacrene B (9.22%), and *α*-bisabolol (9.05%) represented the major volatile components in the flower’s HD EO, while in the case of MAHD, humulene (11.02%), *β*-caryophyllene (8.18%), and *α*-bisabolol (8.03%) were the most abundant volatiles. However, *β-*caryophyllene (14.99%), humulene(7.02%), and *α*-bisabolol (7.65%) represented the major volatile components in SFE. These values were reflected in the total percentage of non-oxygenated volatile compounds, which in all methods, showed high percentages (61.76%, 50.42%, and 51.76% in HD, MAHD, and SEF, respectively) than the oxygenated compounds (25.79% (HD), 31.54% (MAHD), and 16.7% (SEF)). Moreover, the percentage of monoterpene hydrocarbons (MH) is nearly the same in HD and MAHD (5.29%–5.79%), while completely absent in SFE samples. Interestingly, the sesquiterpene hydrocarbons (SH) represented the major percentage in HD (56.47%), followed by MAHD (44.63%) and SEF (38.08%). Also, it was noticed that *β*-sitosterol was detected only in SFE oil with 13.68%, while oxygenated monoterpenes were detected only in MAHD (1.21%). Lastly, OS represents the major percentage in HD (25.79%) and MAHD (30.33%) than that present in SEF oil (16.33%).

**TABLE 2:** Identified compounds of *P*. *cattleianum* flowers’ essential oil extracted by different methods.

Peak	R_t_	Compound	M.F.	RI_exp_	RI_lit_	%Content			Identifications
						HD	MAHD	SFE	
1	7.31	*α*-Pinene	C_10_H_16_	915	915	3.45	2.90	-	MS, RI
2	8.59	*β*-Pinene	C_10_H_16_	961	961	-	0.26	-	MS, RI
3	9.05	*β*-Myrcene	C_10_H_16_	978	978	-	0.21	-	MS, RI
4	9.44	Pseudolimonen	C_10_H_16_	992	996	-	0.05	-	MS, RI
5	10.20	D-Limonene	C_10_H_16_	1017	1027	0.59	0.06	-	MS, RI
6	10.51	*trans-β-*Ocimene	C_10_H_16_	1027	1027	-	0.14	-	MS, RI
7	10.83	*cis-β*-Ocimene	C_10_H_16_	1038	1038	-	0.51	-	MS, RI
8	12.44	*β*-Linalool	C_10_H_18_	1090	1090	1.25	1.66	-	MS, RI
9	12.68	*trans*-Dihydrocarvone	C_10_H_16_O	1097	1193	-	0.07	-	MS, RI
10	13.65	Isopinocarveol	C_10_H_16_O	1129	1136	-	0.04	-	MS, RI
11	14.84	Terpinen-4-ol	C_10_H_18_O	1167	1167	-	0.07	-	MS, RI
12	15.26	*α*-Terpineol	C_10_H_18_O	1180	1180	-	0.25	-	MS, RI
13	18.06	*trans*-Linalool oxide acetate	C_12_H_20_O_3_	1277	1282	-	0.04	-	MS, RI
14	19.85	*α*-Cubebene	C_15_H_24_	1339	1339	-	0.06	-	MS, RI
15	20.05	α-Longipinene	C_15_H_24_	1346	1347	-	0.05	-	MS, RI
16	20.37	(+)-Cyclosativene	C_15_H_24_	1358	1358	-	0.06	-	MS, RI
17	20.49	Ylangene	C_15_H_24_	1362	1362	-	0.13	-	MS, RI
18	20.59	Copaene	C_15_H_24_	1365	1365	1.85	1.95	0.64	MS, RI
19	20.97	Geranyl acetate	C_12_H_20_O_2_	1372	1372	-	0.74	-	MS, RI
20	21.42	7-epi-Sesquithujene	C_15_H_24_	1394	1391	-	0.18	-	MS, RI
21	21.54	*α*-Gurgujene	C_15_H_24_	1398	1398	0.19	-	-	MS, RI
22	21.56	*β*-Maaliene	C_15_H_24_	1399	1381	-	0.32	-	MS, RI
23	21.64	cis-*α*-Bergamotene	C_15_H_24_	1402	1403	0.25	-	-	MS, RI
24	21.82	*β*-Caryophyllene	C_15_H_24_	1409	1409	18.85	8.18	14.99	MS, RI
25	22.33	Aromandendrene	C_15_H_24_	1429	1429	0.61	0.76	0.57	MS, RI
26	22.73	*α*-Humulene	C_15_H_24_	1445	1445	14.46	11.02	7.21	MS, RI
27	22.95	Alloaromadendrene	C_15_H_24_	1452	1452	0.38	0.49	0.75	MS, RI
28	23.31	*γ*-Muurolene	C_15_H_24_	1467	1467	0.59	1.09	0.59	MS, RI
29	23.45	Germacrene D	C_15_H_24_	1473	1473	-	0.97	0.24	MS, RI
30	23.59	Eudesma-4(14),11-diene	C_15_H_24_	1478	1478	0.91	3.74	0.55	MS, RI
31	23.84	*β*-Cyclogermacrane	C_15_H_24_	1488	1488	-	-	1.84	MS, RI
32	24.09	*β*-Bisabolene	C_15_H_24_	1498	1498	1.31	2.07	1.14	MS, RI
33	24.16	*β*-Curcumene	C_15_H_24_	1501	1504	0.57	-	-	MS, RI
34	24.28	cis-*γ*-Bisabolene	C_15_H_24_	1505	1507	1.06	1.09	0.75	MS,RI
35	24.50	Cadina-1(10),4-diene	C_15_H_24_	1514	1514	3.11	3.07	2.15	MS, RI
36	24.57	Cadina-3,9-diene	C_15_H_24_	1517	1518	-	3.07	-	MS, RI
37	24.83	Eudesma-4(14),7(11)-diene	C_15_H_24_	1527	1544	1.47	1.92	0.92	MS, RI
38	25.00	Selina-3,7(11)-diene	C_15_H_24_	1533	1532	1.64	1.92	1.44	MS, RI
39	25.41	Germacrene B	C_15_H_24_	1549	1549	9.22	2.49	4.30	MS, RI
40	25.91	Spatulenol	C_15_H_26_O	1569	1569	-	-	0.32	MS, RI
41	26.07	Caryophyllene oxide	C_15_H_26_O	1575	1575	3.42	-	-	MS, RI
42	26.29	Viridiflorol	C_15_H_26_O	1584	1584	0.59	-	0.2	MS, RI
43	26.56	Ledol	C_15_H_26_O	1594	1595	-	0.16	0.69	MS, RI
44	26.57	(-)-Globulol	C_15_H_26_O	1594	1580	1.23	-	1.75	MS, RI
45	26.71	Humulene 6,7-epoxide	C_15_H_26_O	1600	1600	1.07	2.14	0.24	MS, RI
46	27.13	Di-*epi-*1,10-cubenol	C_15_H_26_O	1619	1619	1.99	2.97	0.98	MS, RI
47	27.26	Cis-2,3,4,4a,5,6,7,8-octahydro-1,1,4a,7-tetramethyl-, 1H-benzocyclohepten-7-ol	C_15_H_26_O	1624	1616	1.09	-	-	MS, RI
48	27.34	Acorenone B	C_15_H_24_O	1624	1620	-	0.85	-	MS, RI
49	27.47	*tau*-Muurolol	C_15_H_26_O	1633	1634	1.21	2.27	-	MS, RI
50	27.52	*α*-Cadinol	C_15_H_26_O	1635	1635	2.36	4.07	1.50	MS, RI
51	27.78	Neointermedeol	C_15_H_24_O	1646	1656	-	4.07	1.5	MS, RI
52	28.09	*cis*-Sesquisabinene hydrate	C_15_H_26_O	1660	1590	1.84	2.56	0.89	MS, RI
53	28.40	*α*-Bisabolol	C_15_H_26_O	1673	1673	9.05	8.03	7.65	MS, RI
54	28.77	Eudesm-7(11)-en-4-ol	C_15_H_26_O	1690	1690	1.94	0.86	0.98	
55	29.26	Farnesol	C_15_H_26_O	1711	1711	-	1.66	-	
56	30.04	*α*-Cyperone	C_15_H_22_O	1745	1755	-	0.07	-	MS, RI
57	30.40	6-Isopropenyl-4,8a-dimethyl-1,2,3,5,6,7,8,8a-octahydro-naphthalen-2-ol	C_15_H_26_O	1760	1714	-	0.29	-	MS, RI
58	31.81	*trans*-Farnesyl acetate	C_17_H_28_O_2_	1824	1824	-	0.33	-	MS, RI
59	31.98	*β*-Sitosterol	C_29_H_50_O	3277	3203	-	-	13.68	MS, RI
		Total identified compounds				87.55	81.96	68.46	
		Non-oxygenated							
		Monoterpene hydrocarbons (MH)				5.29	5.79	-	
		Sesquiterpene hydrocarbons (SH)				56.47	44.63	38.08	
		Sterols				-	-	13.68	
		Oxygenated							
		Oxygenated monoterpenes (OM)				-	1.21	-	
		Oxygenated sesquiterpenes (OS)				25.79	30.33	16.7	

R_t_, retention time; RI_exp_, experimental refractive index; RI_lit_, reference refractive index; M.F, molecular formula.

From the analysis of our results, it was found that *β* caryophyllene, humulene, germacrene B, and *α*-bisabolol (Supplementary Figure S7) represented the major components in both leaves and flowers’ EOs but with variable percentages based either on the investigated organ or the preparation method. Moreover, the SEF oil of the flower showed a high percentage of *β*-sitosterol. Yet, in comparison to the previously published data about *P. cattleianum,* it was found that *β-*caryophyllene represents the major compound in most HD-based reports about leaves’ EOs (Soliman et al., 2016), while caryophyllene oxide (Chalannavar et al., 2013) was detected as a major compound in few reports. Conclusively, the qualitative and quantitative variations in the essential oils derived from the leaves and flowers of *P. cattleianum* compared to other prior studies may be attributed to various factors, such as genetic variations, environmental conditions, harvesting time, drying period, or extraction temperature (Patel et al., 2016).

### 3.2 Biological activity

As it was revealed from the EO yield extracted by the three methods, it was found that SFE offered the highest yield being 0.62% and 1.4% from the leaves and flowers, respectively, so it was selected for further biological assessment.

#### 3.2.1 *In vitro* anti-inflammatory activity

The lipoxygenase (LOX) pathway is the main source of potent proinflammatory leukotrienes (LTs) supplied from arachidonic acid metabolism (AA). Therefore, its inhibition can help with anti-inflammatory effects (Hu and Ma, 2017). In the current study, the EOs from the leaves and flowers of *P. cattleianum* cultivated in Egypt were screened against the 5-LOX enzyme, and the results (Table 3) showed that leaves’ EOs exerted a strong 5-LOX inhibitory effect with IC_50_ 2.380 μL/mL, while the flowers’ EOs displayed weak activity (IC_50_, 7.697 μL/mL) in comparison with the positive control, zileuton (IC_50_, 0.423 μM/mL). Other recognized enzymes that significantly mediate the inflammatory response are the cyclooxygenase isozymes COX-1 and COX-2; the first isozyme is constitutively stated in all organs and particularly responsible for the gastrointestinal protection, while the other isozyme is prevailing at inflammation sites (COX-2) (Hawkey, 2001). In the current investigation, both organs’ SFE oils were screened for their COX inhibitory activities, and the results showed that the leaves’ EOs exerted low COX-1 inhibitory activity (IC_50_ 45.96 μL/mL) in comparison with SC560 (standard, selective COX-1 inhibitor, IC_50_ 0.12 nM), moderate COX-2 inhibitory activity (IC_50_ 9.116 μL/mL) in comparison with indomethacin (IC_50_, 6.653 μg/mL), and finally almost no significant effect in comparison with celecoxib (IC_50_, 0.547 μM/mL). It is noteworthy that the flower’s EOs showed moderate COX-1 activity (IC_50_, 19.08 μL/mL) in comparison with celecoxib (IC_50_, 11.34 μg/mL) and SC560 (IC_50_, 6.34 nM), while high IC_50_ in comparison with indomethacin (IC_50_, 1.067 μg/mL). Moreover, in the case of COX-2, it exhibited strong inhibitory activity (IC_50_, 2.575 μL/mL) as compared to indomethacin (IC_50_, 6.653 μg/mL) and celecoxib (IC_50_, 0.547 μM/mL).

**TABLE 3:** IC_50_ of *P. cattleianum* leaves (L) and flowers’ (F) essential oils against 5-LOX, COX-1, and COX-2 enzymatic activities.

Tested EO	IC_50_ ± SD (µL/mL)		
	5-LOX	COX-1	COX-2
**L**	2.38 ± 0.10	45.96 ± 2.32	9.116 ± 0.25
**F**	7.69 ± 0.22	19.08 ± 0.96	2.575 ± 0.07
**Indomethacin (µg/mL)**	—	1.067 ± 0.05	6.653 ± 0.18
**SC560 (nM)**	—	6.45 ± 0.05	—
**Celecoxib (µM)**	—	11.34 ± 0.57	0.547 ± 0.01
**Zileuton (µM)**	0.42 ± 0.02	-	—-

#### 3.2.2 *In vitro* cytotoxic activity

The Eos of the leaves and flowers of *P. cattleianum* were initially screened for their cytotoxic potential against the available in-house cancer cell lines, namely, MCF-7, HepG2, and K562, and the normal cell line WI38. The results (Table 4) revealed that both organs’ EOs exhibited strong anticancer activity against the three cell lines to a different extent. Yet, the leaves’ oil displayed selective potent growth inhibitory activity to MCF-7 cells (IC_50_, 5.32 μL/mL), followed by K562 (IC_50_, 12.30 μL/mL) and lastly HepG2 (IC_50_, 25.7 μL/mL). However, it showed far low IC_50_ on the normal WI38 cells (IC_50_, 59.7 μL/mL), demonstrating its selectivity to the cancer cell lines, exclusively MCF-7 (SI,11.2), which is better than the SI of doxorubicin (SI, 13.8). On the other hand, the screening of the flowers’ EOs in the same assay demonstrated that K562 (IC_50_, 31.70 μL/mL) is the most sensitive cell line, followed by MCF-7 (IC_50_, 36.20 μL/mL), while HepG2 is the least sensitive cell line to the applied treatment (IC_50_, 58.10 μL/mL). Even though this is the first time in reporting the screening of the flowers’ EOs on various cancer cell lines, the promising, selective anticancer potential of leaves’ EOs was our compelling rationale. Hence, a complete mechanistic study was accomplished to understand the mode of cell death and the proposed molecular targets involved in the observed anticancer potential.

**TABLE 4:** IC_50_ of *P. cattleianum* leaves (L) and flowers’ (F) essential oils against cancer cell lines (MCF‐7, HepG2, K562) and normal cell line (WI38).

Tested EO	IC_50_ ± SD (µL/mL)			
	Cancer cell lines			Normal cells
	MCF-7	HepG2	K562	WI38
L	5.32 ± 0.29	25.70 ± 1.38	12.30 ± 0.66	59.70 ± 3.22
F	36.20 ± 1.95	58.10 ± 3.13	31.70 ± 1.71	43.40 ± 2.34
Doxorubicin (µg/mL)	4.46 ± 0.24	7.5 ± 0.4	0.72 ± 0.04	13.8 ± 0.74

#### 3.2.3 Cell cycle analysis and detection of apoptosis in MCF-7 cells

**FIGURE 1:**
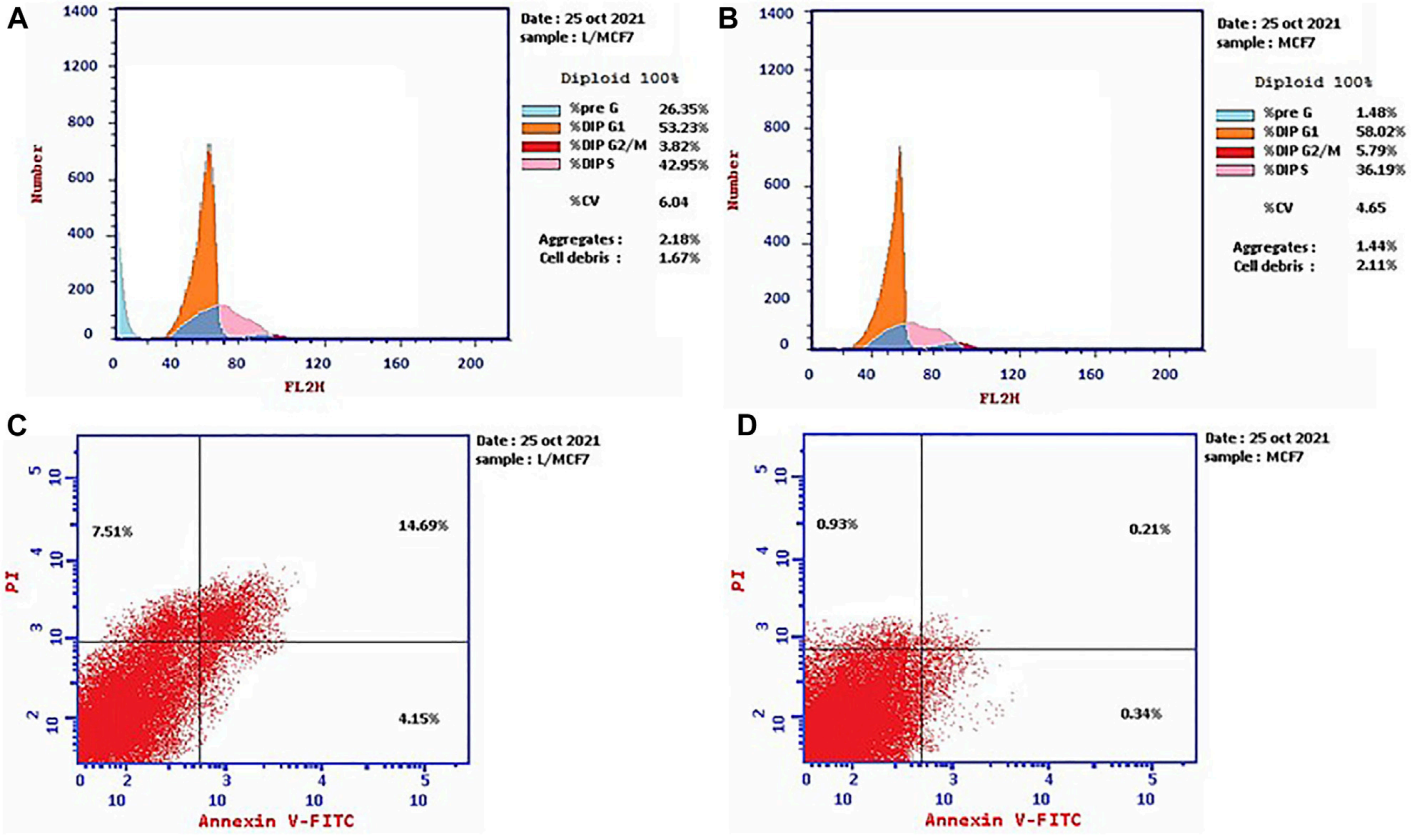
Cell cycle distributions of MCF-7 cells **(A)** treated with EOs obtained from *P. cattleianum* Sabine leaves compared to **(B)** untreated, control cells. Percentage of early, late apoptotic, and necrotic cells in MCF-7 cells **(C)** treated with EOs obtained from *P. cattleianum* leaves compared to **(D)** untreated, control.

Propidium iodide (PI) is commonly used in combination with annexin V to measure if the cells are viable, apoptotic, or necrotic *via* observing the changes in the integrity and permeability of the plasma membrane (Rieger et al., 2011). The intact cell and nuclear membranes inhibit PI entrance; hence, they do not stain either live or early apoptotic cells; on the other hand, in late apoptotic and necrotic cells, the integrity of the plasma and nuclear membranes is reduced, allowing PI to permit and intercalate into nucleic acids, and revealed red fluorescence (Rieger et al., 2011). In this study, the MCF-7 cells, treated with the leaves’ EOs, were subjected to cell cycle analysis to gain understanding of the cytotoxicity mechanism and mode of cell death. The results (Figure 1, Supplementary Table S1) demonstrated that the cell percentage in the G_0_–G_1_ and G_2_/M phases decreased upon treatment, being 53.23% and 3.82%, respectively, in comparison with the untreated control group. Moreover, it exhibited an increase in the cellular population of the S and pre-G_1_ phases to 42.95% and 26.35% compared to the control (36.19% and 1.48%, respectively). Subsequently, the apoptosis percentage increased from 1.48% in the control cells to 26.35%, whereas the percentage of necrotic cells increased to 7.51% compared to 0.93% in untreated cells (Figure 1, Supplementary Table S1).

#### 3.2.4 Expression of apoptosis-related proteins using the Western blot technique

**FIGURE 2:**
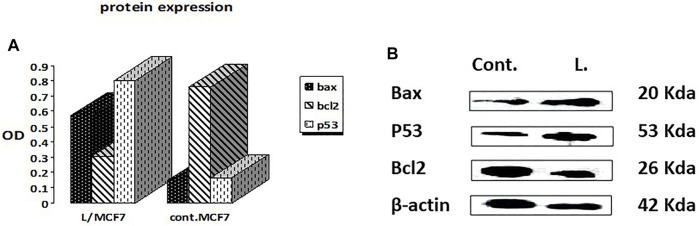
Effects of treatment by the EOs obtained from *P. cattleianum* Sabine leaves on the protein expression levels of **(A)** p53, Bax, Bcl-2, and *β*-actin; **(B)** protein relative abundance as measured from band intensity compared to *β*-actin.

The Western blotting technique was adopted to quantify the total levels of the apoptosis-mediated proteins, namely, Bax, Bcl2, and p53 (Mahmood and Yang, 2012). The results showed that the leaves’ EOs significantly overexpressed the Bax and p53 protein levels by 3.9 and 4.8 folds, respectively, in comparison with the control and untreated group, while the level of the Bcl-2 protein level was decreased to about 0.4 in the control (Figure 2, Supplementary Figure S8). Therefore, we expect that leaves’ EOs modulated the apoptotic pathway by regulating the p53-Bax/Bcl2 axis in MCF-7. Several reports were conducted on the biological activities of *cattleianum* leaves’ EOs, which revealed its significant antioxidant, antifungal, antibacterial (Castro et al., 2014), and anticancer activities against HeLa (human cervical adenocarcinoma cells), HepG2, AGS (human gastric cancer cells), SNU-1 (colorectal cancer cells), and SNU-16 (human stomach cancer cells) (Fidyt et al., 2016). Meanwhile, there are no studies about the anti-inflammatory and anticancer activities of flower-derived EOs and the anti-inflammatory and anticancer activities of leaves’ EOs, especially against MCF-7 and K562. The chemical components of leaves and flowers’ EOs prepared by the SFE method are responsible for their biological activities. It was found that both oils pioneered principally with *β*-caryophyllene (BCP), representing 13.2% and 14.99% for leaves and flowers, respectively, together with *α*-humulene (*α*-caryophyllene), which represented 10.9% (leaves) and 7.21% (flower). Moreover, the leaves’ EOs are also traced with caryophyllene oxide (BCPO, the oxidation derivative of *β*-caryophyllene) being 4.81%, which is almost absent from the flowers' EOs. BCPO is one of the major active components in various EOs derived from numerous food and spices. It possessed different biological effects such as anti-inflammatory (Medeiros et al., 2007) and anticarcinogenic (Langhasova et al., 2014) effects. BCP belongs to cannabinoid compounds (CBS), especially phytocannabinoids. However, cannabinoids could stimulate the cannabinoid receptors (CB_1_ and CB_2_), while BCP activates only CB_2_ and has no affinity to CB_1_, which explains that BPC action is lacking psychoactive side effects allied with cannabinoids and recommends its potential use in medicine (Fidyt et al., 2016). Moreover, *α*-humulene and BCPO have no affinity to CB_1_ and CB_2_, which explain that both compounds exhibited their biological activities through partially different mechanisms such as apoptosis induction, repression of the cell cycle, and inhibition of angiogenesis and metastasis (Carracedo et al., 2006). Many investigations have been performed to unravel the anticancer mechanism of BCPO, while that of BCP has hardly been studied (Fidyt et al., 2016). Many reports have mentioned the strong anti-proliferative activity of BCP against many cell lines due to its antiangiogenic properties, which are attributed to its interaction with the hypoxia-inducible transcription factor-1alpha (HIF-1α) that controls the biological pathways associated with hypoxia, tumor metastasis, tumor-mediated angiogenesis, and vascular endothelial growth factor transcription (VEGF) (Ghosh et al., 2022). Moreover, it was reported that BCPO possessed methylene and epoxide exocyclic functional groups, which bind covalently to the DNA nitrogenous bases and proteins by sulfhydryl and amino groups. Thus, BCPO exhibited great potential as a signaling modulator in tumor cancer cells (Park et al., 2011). Other reports revealed that BCPO has anticancer effects on MCF-7 and prostate cancer cell lines through the indication of ROS generation, MAPK activation, and inhibition of the PI3K/AKT/mTOR/S6K1 signaling pathway, which is vital to cell survival, proliferation, and angiogenesis of the tumor (Lo Piccolo et al., 2008). Additionally, it significantly decreases key protein levels involved in proliferation (cyclinD1), metastasis, angiogenesis (VEGF), and apoptosis inhibitors Bcl-2 (B-cell lymphoma 2) and IAP-1/2 (inhibitor of apoptosis 1 and 2) (Ryu et al., 2012). Moreover, it was reported that BCOP can exert pro-apoptotic activity in cancer cells through a reduction in NF-κB as a key transcription factor in the development of tumors through monitoring cancer cell proliferation, tumorigenesis, angiogenesis, and metastasis (Sain et al., 2014), and it regulated several genes implicated in cellular proliferation, apoptosis, and inflammation. Furthermore, it was reported that *α*-humulene and BCPO exhibited significant anti-proliferative activities against different cell lines witnessed by their combination with BCP in decreasing MCF-7 proliferation compared to when used separately (Legault et al., 2007). An observation that is in good agreement with our results is that leaves’ EOs exerted more potent cytotoxic effects than flowers’ EOs which may be due to the absence of BCPO from the flower. Moreover, it was stated that there is a possibility of a synergistic effect between the volatile components in EOs instead of only one major component or isolated compounds in modulating the cancer pathway. Additionally, it was reported that the strength of the cellular response induced after treatment with BCP(O) compounds differs significantly among cancer cells, which was also figured out from our results.

## 4 Conclusion

Essential oils (EOs) from *P. cattleianum* leaves and flowers, cultivated in Egypt, have been extracted by three different methods and evaluated in terms of their chemical composition and biological significance. The implemented extraction methods greatly affected the yield, in addition to the qualitative and quantitative chemical properties of the EOs. Supercritical fluid extraction (SFE), represented an environment-friendly method and offered the highest EO yield from both organs with minimal degradation drawbacks, while the least yield was indicated by the conventional hydro-distillation method. *P. cattleianum* EOs were superlative by terpenoid hydrocarbons such as *α*-humulene, *β*-caryophyllene, and germacrene B, while *α*-bisabolol and caryophyllene oxide represented the major, identified oxygenated terpenes. The leaves’ EOs showed potent, anti-inflammatory capacity *via* inhibiting the 5-LOX enzyme, while the flowers inhibited the COX-2 enzyme. In addition, the leaves’ EOs induced apoptosis in the MCF-7 breast cancer cell line by modulating the P53-Bax/Bcl2 axis. The observed promising activities is, at least in part, due to the synergism between the volatile components; hence, *P. cattleianum*-derived EOs may be promoted as dietary supplements for the management of breast malignancies and inflammatory disorders.

## Data Availability

The original contributions presented in the study are included in the article/Supplementary Materials; further inquiries can be directed to the corresponding author.
