# Corrigendum: Essential oils of *Psidium cattleianum* Sabine leaves and flowers: anti-inflammatory and cytotoxic activities

**DOI:** 10.3389/fchem.2023.1213446

**Published:** 2023-06-19

**Authors:** Heba E. Elsayed, Eman M. El-Deeb, Heba Taha, Hussein S. Taha, Mohamed R. Elgindi, Fatma A. Moharram

**Affiliations:** ^1^ Pharmacognosy Department, Faculty of Pharmacy, Helwan University, Cairo, Egypt; ^2^ Pharmacognosy Department, Faculty of Pharmacy, October 6 University, Giza, Egypt; ^3^ Biochemistry and Molecular Biology Department, Faculty of Pharmacy, Helwan University, Cairo, Egypt; ^4^ Department of Plant Biotechnology, Genetic Engineering and Biotechnology Division, National Research Centre, Cairo, Egypt

**Keywords:** anti-inflammatory, caryophyllene, cytotoxicity, MCF-7, *Psidium cattleianum*, supercritical fluid extraction

In the published article, there was an error in author **Affiliation 4**. Instead of “Department of Plant Biotechnology, Genetic Engineering Division, Cairo, Egypt,” it should be “Department of Plant Biotechnology, Genetic Engineering Division, National Research Centre, Cairo, Egypt.”

The authors apologize for this error and state that this does not change the scientific conclusions of the article in any way. The original article has been updated.

