# Supplementary material for: Essential oils of Psidium cattleianum Sabine leaves and flowers: Anti-inflammatory and cytotoxic activities
Source: Front Chem. 2023 Feb 6;11:1120432. doi: 10.3389/fchem.2023.1120432 (PMC9940317; doi:10.3389/fchem.2023.1120432)
Supplement: Supplementary file 1 [file DataSheet1.pdf]

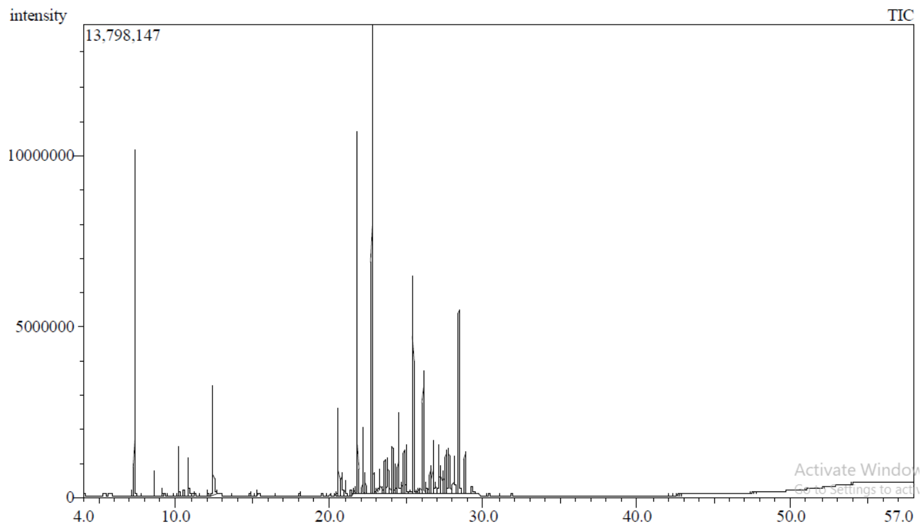

Fig. S1: GC/MS Chromatogram for *P. cattleianum* Sabine leaves essential oil extracted by HD

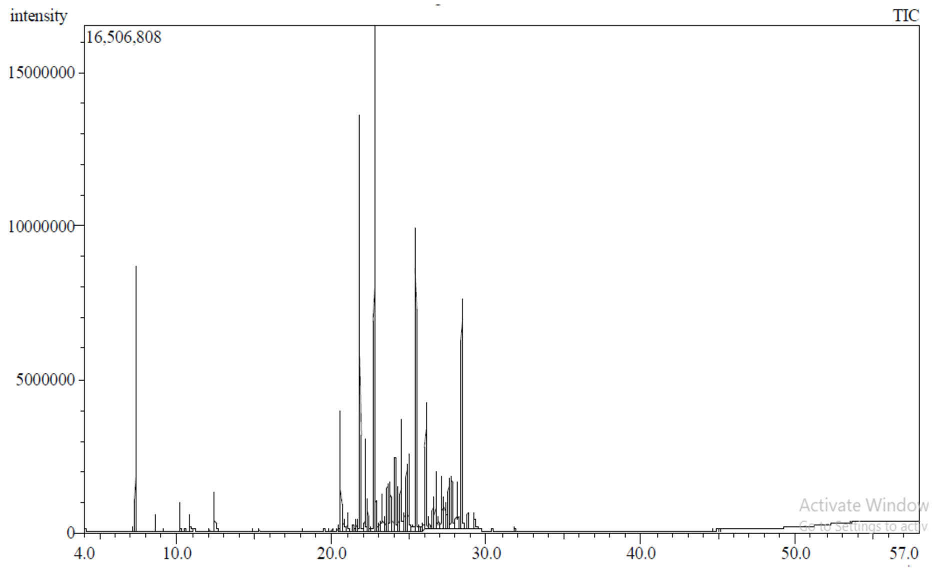

Fig. S2: GC/MS Chromatogram for *P. cattleianum* Sabine leaves essential oil extracted by MAHD

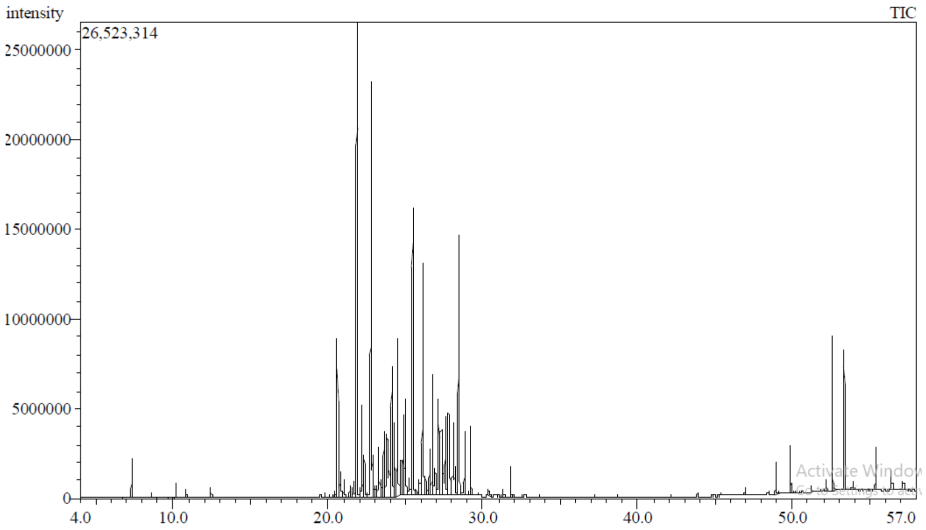

Fig. S3: GC/MS Chromatogram for *P. cattleianum* Sabine leaves essential oil extracted by SFE

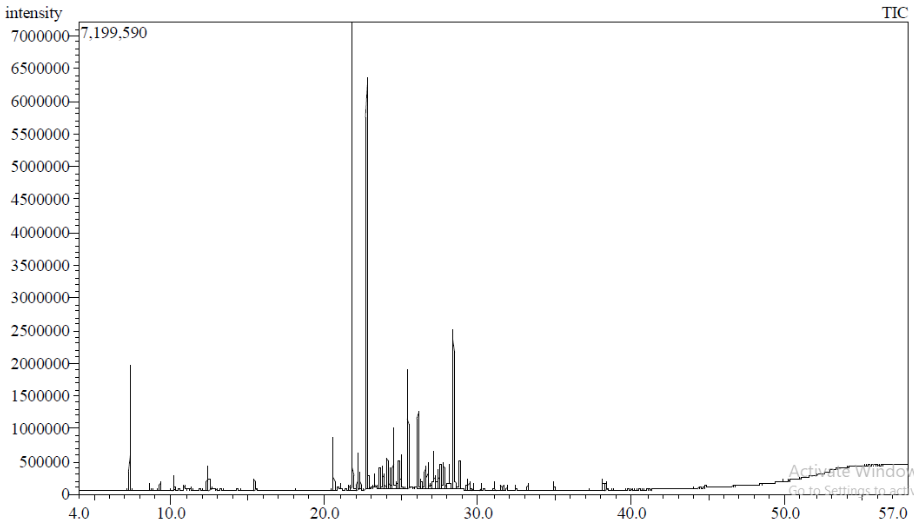

Fig. S4: GC/MS Chromatogram for *P. cattleianum* Sabine flower essential oil extracted by HD

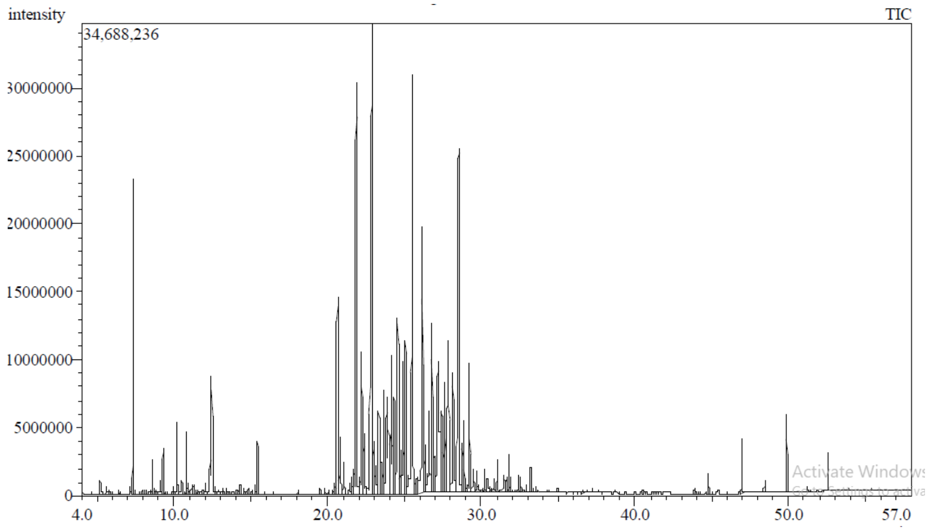

Fig. S5: GC/MS Chromatogram for *P. cattleianum* Sabine flower essential oil extracted by MAHD

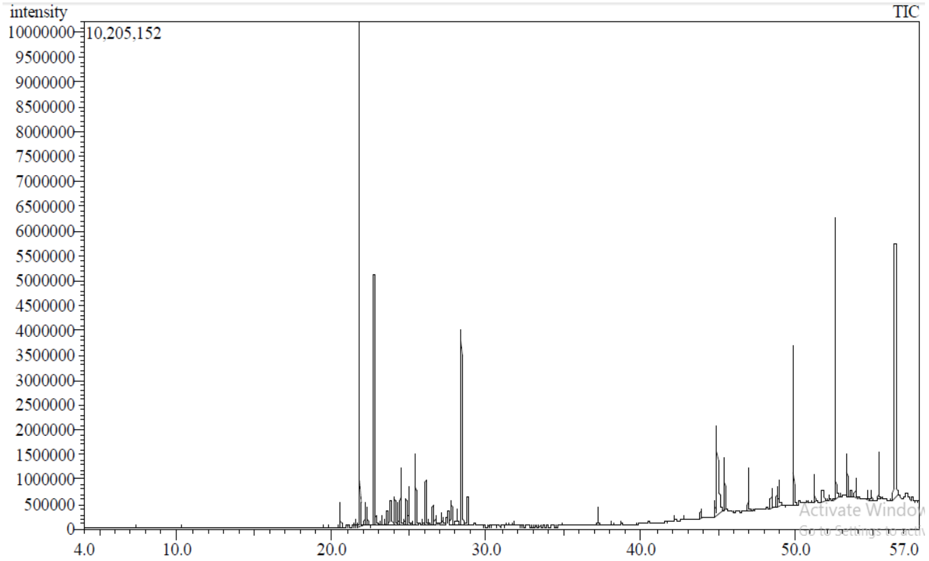

Fig. S6: GC/MS Chromatogram for *P. cattleianum* Sabine flower essential oil extracted by SFE

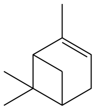

***α*-pinene**

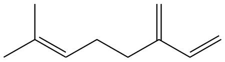

***β*-Myrcene**

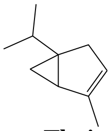

***α*-Thujene**

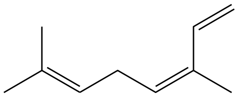

***cis*-*β*-Ocimene**

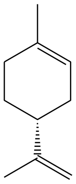

D-limonen

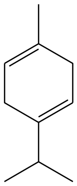

γ-Terpinene

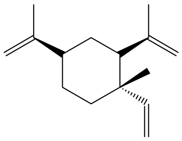

***β*-Elemene**

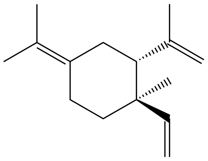

***α*-Elemene**

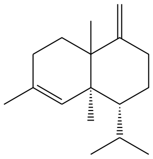

γ-Muurolene

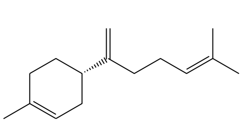

***β*-Bisabolene**

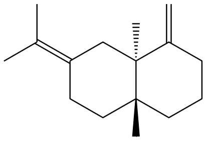

γ –Selinene

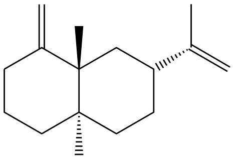

Eudesma-4(14),11-diene= *β* – Selinene

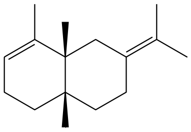

Selina-3,7(11)-diene

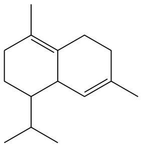

Cadina-1(10),4-diene

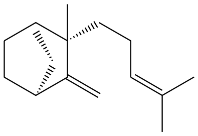

***β*-santalene**

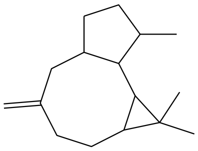

Aromandendrene

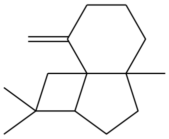

***β*-Panansinene**

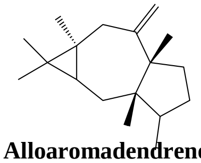

Alloaromadendrene

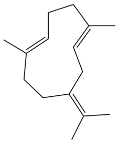

Germacrene B

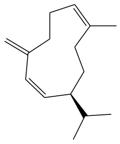

Germacrene D

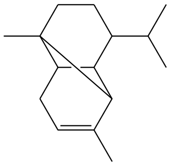

***α*-Copaene**

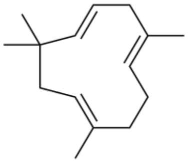

***α*-Humulene**

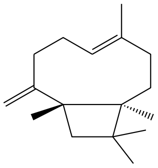

***β*-Caryophyllene**

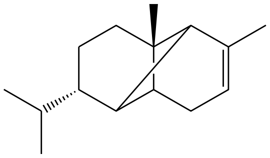

Ylangene

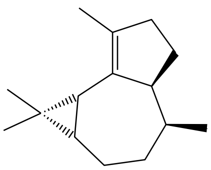

***α*-Gurjunene**

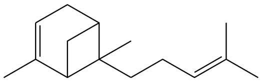

***trans*-*α*-Bergamotene**

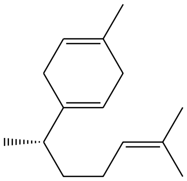

***β*-Curcumene**

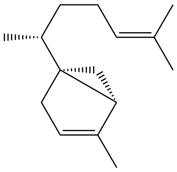

7-*epi*-Sesquithujene

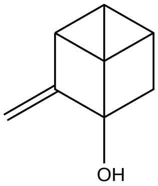

Isopinocarveo

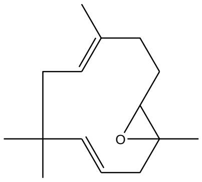

Terpinen-4-ol

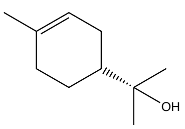

***α*-Terpineol**

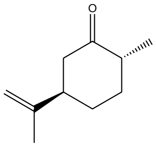

***trans*-Dihydrocaryone**

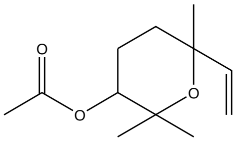

***trans*-Linalool oxide acetate**

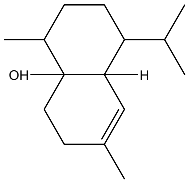

Di-epi-1,10-cubenol

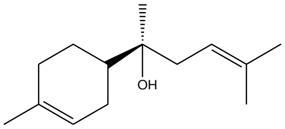

***α*-Bisabolol**

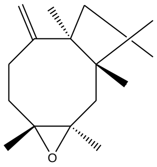

Caryophyllene oxide

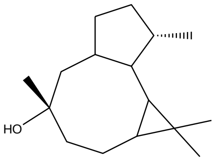

Ledol

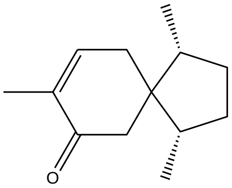

Acorenone B

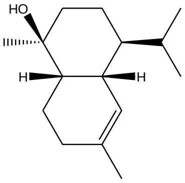

τ-Muurolol

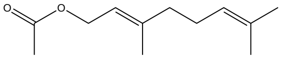

Geranyl acetate

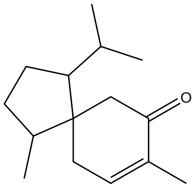

Humulene epoxid I

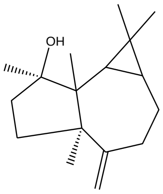

Humulene epoxid II

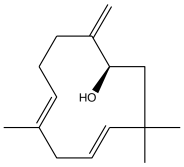

Humulenol II

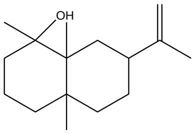

Neointermedeol

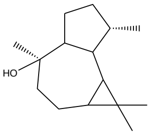

Viridiflorol

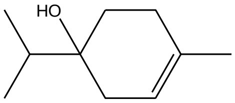

Spathulenol

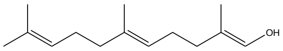

Farnesol

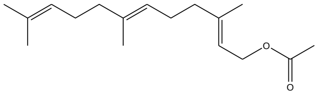

***trans*-Farnesol acetate**

Fig. S7. Structures of the compounds detected in the HD, MAHD and SFE oil samples extracted from the leaves and flowers of *P. cattleianum* Sabine

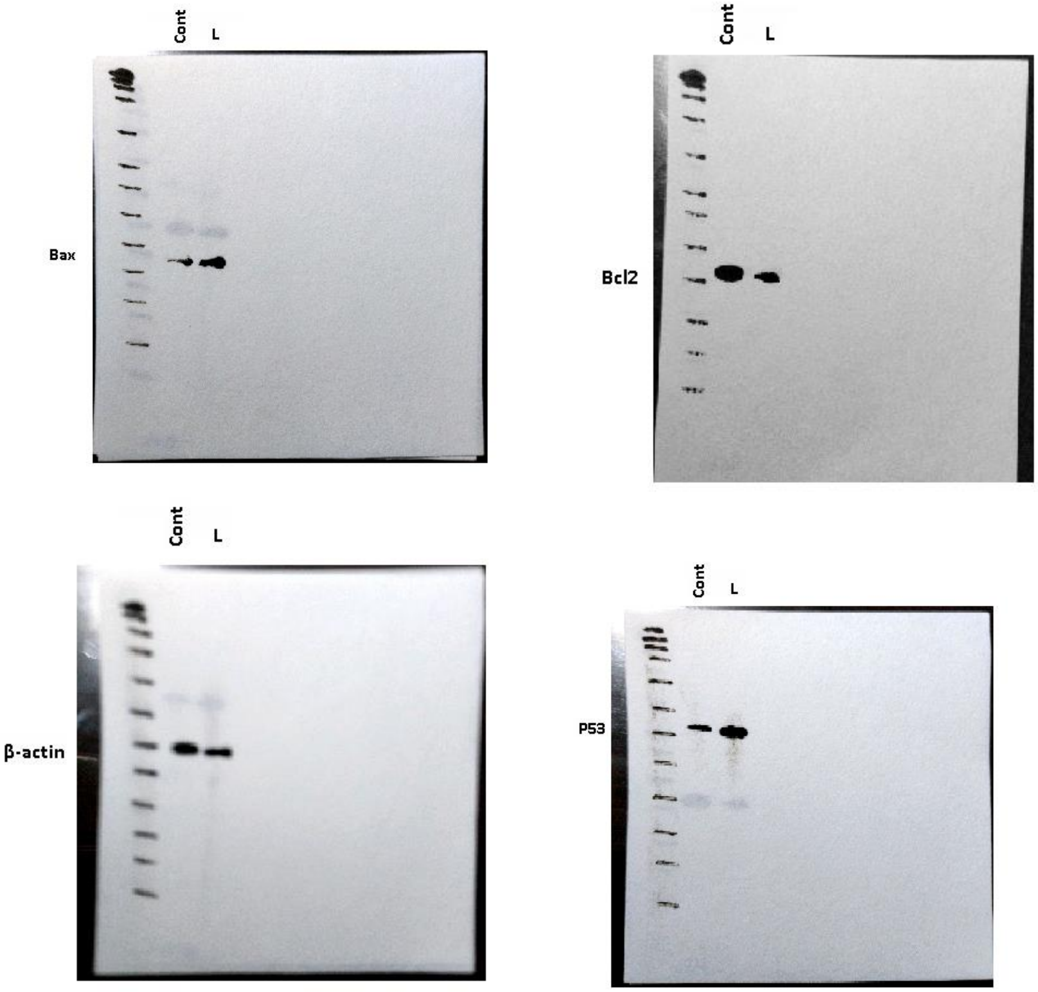

Fig S8: Uncropped, untouched full original image of western blot presenting p53, Bax, Bcl2 and B-actin

Table S1: Percentage of DNA content, early, late apoptotic, and necrotic cells in MCF-7 cells treated with the leaves EO versus control non-treated MCF-7 cells

	G_0-G_1 %	S %	G_2/M %	Pre-G_1%	Apoptosis %			Necrosis %
					Total	Early	Late	
**Control cells**	58.02	36.19	5.79	1.48	1.48	0.34	0.21	0.93
**Treated cells**	53.23	42.95	3.82	26.35	26.35	4.15	14.69	7.51
